# Intravenous magnesium sulphate for aneurysmal subarachnoid hemorrhage: an updated systemic review and meta-analysis

**DOI:** 10.1186/cc10017

**Published:** 2011-02-07

**Authors:** George KC Wong, Ronald Boet, Wai S Poon, Matthew TV Chan, Tony Gin, Stephanie CP Ng, Benny CY Zee

**Affiliations:** 1 Division of Neurosurgery, Prince of Wales Hospital, The Chinese University of Hong Kong, 30-32 Ngan Shing Road, Shatin, NT, Hong Kong SAR, PR China; 2 Surgical Services, St. George's Hospital, 249 Papanui Road, Strowan, Christchurch 8014, New Zealand; 3 Department of Anesthesia and Intensive Care, Prince of Wales Hospital, The Chinese University of Hong Kong, 30-32 Ngan Shing Road, Shatin, NT, Hong Kong SAR, PR China; 4 School of Public Health, Prince of Wales Hospital, The Chinese University of Hong Kong, 30-32 Ngan Shing Road, Shatin, NT, Hong Kong SAR, PR China

## Abstract

**Introduction:**

Previous meta-analyses of magnesium sulphate infusion in the treatment of aneurysmal subarachnoid hemorrhage (SAH) have become outdated due to recently published clinical trials. Our aim was thus to perform an up-to-date systemic review and meta-analysis of published data on the use of magnesium sulphate infusion in aneurysmal SAH patients.

**Methods:**

A systemic review and meta-analysis of the literature was carried out on published randomized controlled clinical trials that investigated the efficacy of magnesium sulphate infusion in aneurysmal SAH patients. The results were analyzed with regard to delayed cerebral ischemia (DCI), delayed cerebral infarction, and favorable neurological outcomes at three and six months. The risks of bias were assessed using the Jadad criteria, with a Jadad score >3 indicating a lower such risk. Meta-analyses are presented in terms of relative risk (RR) with 95% confidence intervals (CIs).

**Results:**

Six eligible studies with 875 patients were reviewed. The pooled RR for DCI was 0.87 (95% CI, 0.36 to 2.09; *P = 0.75*). That for delayed cerebral infarction was 0.58 (95% CI, 0.35 to 0.97; *P = 0.04*), although this result did not persist if only randomized clinical trials with a lower risk of bias were included (RR 0.61, 95% CI, 0.31 to 1.22; *P = 0.17*). The pooled RR for a favorable outcome at three months was 1.14 (95% CI, 0.99 to 1.31; *P = 0.07*), and that for a favorable outcome at six months was 1.08 (95% CI, 0.94 to 1.24; *P = 0.29*).

**Conclusions:**

The present findings do not lend support to a beneficial effect of magnesium sulphate infusion on delayed cerebral infarction. The reduction in DCI and improvement in the clinical outcomes of aneurysmal SAH patients following magnesium sulphate infusion observed in previous pilot studies are not confirmed, although a beneficial effect cannot be ruled out because of sample size limitation.

## Introduction

Although spontaneous subarachnoid hemorrhage (SAH) accounts for only 3 to 5% of all strokes and 4.4% of deaths from stroke [1,2], the relative youth of the individuals affected means that it is actually responsible for approximately 25% of all years of life lost as a result of stroke [3]. Such complications as early brain injury and delayed ischemic neurological deficits remain a major cause of morbidity and mortality in this group of patients.

Pilot clinical trials using magnesium sulphate in patients with acute aneurysmal SAH have reported a trend toward a reduction in clinical deterioration due to delayed cerebral ischemia (DCI) and an improvement in clinical outcomes [4-11], although two recently completed clinical trials failed to demonstrate any improvement in neurological outcomes [12,13]. Interestingly, however, a German trial found a significant decrease in delayed cerebral infarction but no improvement in neurological outcomes, in contrast to other retrospective analyses [13-15].

Previous meta-analyses of the use of magnesium sulphate infusion to treat aneurysmal SAH have become outdated due to recently published clinical trials [16-18]. Hence, we conducted an up-to-date literature review and meta-analysis of published data on patients with DCI, delayed cerebral infarction, or neurological outcomes.

## Materials and methods

### Type of studies

We included only randomized controlled clinical trials comparing magnesium sulphate infusion to placebo infusion for patients with acute aneurysmal SAH.

### Types of outcome measures

The primary outcome was dichotomized neurological outcome using Glasgow Outcome Scale (GOS) [19] and modified Rankin Scale (mRS) [20] at three and six months. We also assessed surrogate outcome with DCI and delayed cerebral infarction according to a recent consensus paper [15].

### Definition of outcome measures

In our systemic review and meta-analysis, DCI was defined as the occurrence of: clinical deterioration, which was manifested clinically as a new focal neurological deficit (motor or speech deficit) that developed after SAH or a decrease in the Glasgow Coma Scale of two or more points for more than six hours; and/or delayed cerebral infarction, which was defined as a new cerebral infarction within three weeks that was not related to post-treatment (coiling or clipping) complications, the ventricular catheter track, a rebleed, or hydrocephalus [15].

Another outcome, delayed cerebral infarction, was similarly defined as a new cerebral infarction within three weeks that was not related to post-treatment (coiling or clipping) complications, the ventricular catheter track, a rebleed, or hydrocephalus.

Neurological outcomes were defined by the GOS [19] and mRS [20]. A favorable outcome was defined as a GOS score of 4 to 5 or a mRS score of 0 to 2.

### Search strategy

Cochrane Central Register of Controlled Trials (Clinical Trials), EMBASE, PubMed, and Ovid MEDLINE searches (using the keywords magnesium AND subarachnoid hemorrhage) of studies employing randomized controlled trials and published between 1 January, 1980 and 15 June, 2010 were carried out. The references listed in these publications were also searched for relevant studies.

### Risk of bias assessment

The Jadad criteria were used to assess the risk of bias [21]. The criteria included randomization, blinding, and an explanation of withdrawal or loss to follow up. Clinical trials with Jadad scores of 3 or above were considered to be of high quality. No funnel plot was employed to test for asymmetry, because fewer than 10 clinical trials were ultimately included.

### Statistical analysis

Statistical analyses were generated using SPSS for Windows Version 15.0 (SPSS Inc., Chicago, Illinois, USA) and Review Manager 5 (Cochrane Collaboration, Oxford, UK). Statistical significance was taken as a *P *value less than 0.05 or a 95% confidence interval (CI) of relative risk (RR) not including 1. Data are represented using numbers (percentages) for the categorical variables and mean +/- standard deviations for the numerical variables. The I^2 ^value for heterogeneity describes the proportion of total variation in a study estimated to be due to heterogeneity. The random-effects model was employed to pool studies when statistical heterogeneity occurred (the *P *value was less than 0.1) or when the I^2 ^value was larger than 0.3; otherwise, the fixed-effects model was used. Results are presented using the RRs and 95% CIs.

## Results

### Search results

A PubMed literature search (using the keywords magnesium AND subarachnoid hemorrhage) of studies employing randomized controlled trials and published between 1 January, 1980 and 15 June, 2010 yielded 20 publications. Additional searches of the Cochrane Central Register of Controlled Trials (Clinical Trials), EMBASE, and Ovid MEDLINE (using the keywords magnesium AND subarachnoid hemorrhage and considering only human studies) yielded 51, 72, and 91 publications, respectively. The references listed in these publications were also searched for relevant studies. Examination of the abstracts and/or manuscripts revealed seven completed randomized controlled clinical trials on the use of magnesium in patients with aneurysmal SAH [6,8-13] and one methodology-only abstract [22]. None of these studies detected a statistically significant improvement in primary clinical outcome measures.

### Study descriptions

Veyna and colleagues [6] reported the results of a 40-patient prospective single-blinded clinical trial of high-dose magnesium sulphate infusion therapy (a bolus of 6 g followed by 2 g per hour intravenous infusion, with a target magnesium level of 4 to 5.5 mg/dl) following spontaneous SAH. They enrolled patients with Hunt and Hess grades II to IV at admission and presenting within 72 hours after spontaneous SAH. In the magnesium treatment group, they maintained the magnesium sulphate infusion for 10 days. They presented no data on DCI or delayed cerebral infarction. A favorable outcome (good recovery or moderate disability, as defined by GOS 4 to 5) was achieved in 13 of 20 (65%) patients receiving magnesium sulphate infusion and 10 of 20 (50%) patients receiving the placebo treatment. No data were available for excellent outcome (good recovery, as defined by GOS 5 or mRS 0 to 1).

Van den Bergh and colleagues [8] reported the magnesium group results for the Magnesium and Acetylsalicylic Acid in Subarachnoid Hemorrhage (MASH) trial, a randomized, double-blinded, placebo-controlled multicenter trial with a factorial design. The salicylic acid-related data were not complete at this stage. A total of 283 patients were randomized within four days of aneurysmal SAH. Magnesium treatment consisted of a continuous intravenous dose of 64 mmol/day, begun within four days of SAH and continued until 14 days after occlusion of the aneurysm. CT hypointensities (with clinical features of a decreased consciousness level or new focal neurological deficit) occurred in 22 of 139 (16%) magnesium-treated patients and 35 of 144 (24%) placebo-treated patients, but no data were made available for DCI and delayed cerebral infarction according to the recent consensus paper [15]. Unfavorable outcomes occurred in 38 of 139 (27%) magnesium-treated patients and 51 of 144 (35%) placebo-treated patients. There were no separate outcome data on excellent outcomes.

In our single-center pilot study [9], 60 patients were randomly allocated to receive either magnesium sulphate infusion (80 mmol/day) or saline infusion for 14 days. We did not record DCI or cerebral infarction as a separate outcome measure during this pilot study. A favorable outcome was achieved in 20 of 30 (67%) patients receiving magnesium sulphate infusion and 16 of 30 (53%) patients receiving the placebo treatment, and an excellent outcome in 14 of 30 (47%) of the former and 10 of 30 (33%) of the latter.

Muroi and colleagues [11] reported the results of a prospective, randomized, patient-blinded, placebo-controlled pilot study of 58 patients with aneurysmal SAH predominantly treated by microsurgical clipping (97%). The patients allocated to the treatment group received a bolus of 16 mmol magnesium sulphate administered over 15 minutes, followed by a continuous intravenous infusion of 64 mmol per day for 14 days. To maintain the serum magnesium level at twice the baseline, with a maximum of 2.0 mmol/L until the 14th day after SAH, subsequent dosage adjustments were made every 12 hours. Delayed cerebral infarction occurred in 3 of 31 (10%) patients in the treatment group and 6 of 27 (22%) in the placebo group. A favorable neurological outcome was achieved in 20 of 31 (64%) patients in the treatment group and 13 of 27 (48%) in the placebo group, and an excellent outcome was achieved in 18 of 31 (58%) in the former and 12 of 27 (44%) in the latter.

The Asian-Australasian Intravenous Magnesium Sulphate for Aneurysmal Subarachnoid Hemorrhage (IMASH) trial was a randomized, double-blinded, placebo-controlled, multicenter phase III trial [12], under the protocol of which patients who were diagnosed with acute aneurysmal SAH (within 48 hours of ictus) were randomly assigned to receive either intravenous magnesium sulphate infusion or normal saline infusion (placebo). For patients receiving the active treatment, 20 mmol of magnesium sulphate was administered over 30 minutes, followed by a continuous infusion of 80 mmol of magnesium sulphate per day for up to 14 days after the hemorrhage. The plasma magnesium concentration was measured regularly. The infusion was adjusted to raise the plasma magnesium concentration to approximately twice the baseline value and less than 2.5 mmol/L. The patients in the control group received an equivalent volume of normal saline. The proportions of patients with a favorable outcome were similar: 108 of 169 (64%) in the magnesium sulphate group and 100 of 158 (63%) in the saline group. So too were those with an excellent outcome: 77 of 169 (46%) in the magnesium sulphate group and 71 of 158 (45%) in the saline group. The proportions of patients with DCI and delayed cerebral infarction were also similar.

Westermaier and colleagues [13] recently reported the results of another single-center, randomized controlled clinical trial. Patients were randomly allocated to receive either magnesium sulphate infusion (with a target level of 2.0 to 2.5 mmol/L) or saline infusion for 14 days. This study reported negative clinical outcomes, but showed a reduction in DCI and delayed cerebral infarction with magnesium sulphate infusion. A favorable outcome was reported for 34 of 54 (63%) patients in the treatment group and 27 of 53 (51%) in the placebo group, and an excellent outcome for 27 of 54 (50%) in the former and 18 of 53 (34%) in the latter. DCI occurred in 20 of 53 (37%) patients in the magnesium group and 35 of 53 (66%) in the control group. Delayed cerebral infarction occurred in 12 of 54 (22%) patients in the magnesium group and 27 of 53 (51%) in the control group.

Our pilot study [9], IMASH [12], the magnesium component of the MASH study [8], and the studies published by Veyna and colleagues [6], Muroi and colleagues [11], and Westermaier and colleagues [13] were analyzed. The study by Schmid-Elsasser and colleagues [10] was excluded because of the unconventional omission of nimodipine in the magnesium group. That by Pravedello and colleagues [23] was excluded from the analysis because no measures of DCI, cerebral infarction, or clinical outcomes were reported. The six eligible studies were reviewed according to the terminologies defined in the Methods section (Table 1) The target of the magnesium arm of the six studies was to produce a similar degree of hypermagnesemia, namely, double the baseline value. All of the studies recruited patients with SAH during the acute phase, within 48 to 96 hours of the aneurysmal SAH. The magnesium infusion was maintained for 10 to 14 days, and the neurological outcome was measured using the GOS or mRS at three months [6,8,11] or six months [9,12,13], but not both.

**Table 1:** Outcome parameters of the randomized controlled clinical trials included

Trial	Patient number	Serious adverse events	Delayed cerebral ischemia	Delayed cerebral infarction	Three-month favorable outcome	Three-month excellent outcome	Six-month favorable outcome	Six-month excellent outcome
Veyna 2002 [6]	40	No	No	No	Yes	No	No	No
van den Bergh 2005 [30]	283	Not mentioned	No	No	Yes	No	No	No
Wong 2006 [9]	60	No	No	No	No	No	Yes	Yes
Muroi 2008 [11]	58	Hypotension (12), cardiac events (4)	No	Yes	Yes	Yes	No	No
Westermaier 2010 [13]	107	Hypocalcemic tetany (1)	Yes	Yes	No	No	Yes	Yes
Wong 2010 [12]	327	Limb weakness (1), severe electrolyte disturbance (1)	Yes	Yes	No	No	Yes	Yes

The terminologies for delayed cerebral ischemia, delayed cerebral infarction, and favorable and excellent outcome are defined in the Materials and methods section.

### Assessment of risks of bias in included studies

The six eligible studies were assessed for risks of bias using the Jadad criteria (Table 2). Four of the six trials were found to have lower bias risks (Jadad score above 3) [8,9,12,13]. The authors of this systemic review acknowledged potential risk of bias because we were authors of two of the included studies in this systemic review [9,12].

**Table 2:** Quality assessment of the randomized controlled clinical trials included

Trial	Center	Randomization method	Blind	Explanation for withdrawals	Jadad Scale
Veyna 2002 [6]	Monocenter	Not stated	Patients were blinded	Yes	2
van den Bergh 2005 [30]	Multicenter	Study medications were randomized and distributed by coordinating center	Principal investigators and assessors were blinded	Yes	4
Wong 2006 [9]	Monocenter	Sealed envelopes in order	Assessors and health care staff were blinded	Yes	4
Muroi 2008 [11]	Monocenter	Not stated	Patients were blinded	Yes	2
Westermaier 2010 [13]	Monocenter	Enveloped lot from a box	Assessors were blinded	Yes	4
Wong 2010 [12]	Multicenter	Randomization through Internet/sealed envelopes in order	Patients, assessors, and health care staff were blinded	Yes	4

### Meta-analysis of eligible randomized clinical trials with relevant data available according to the consensus paper definitions

**Figure 1:**
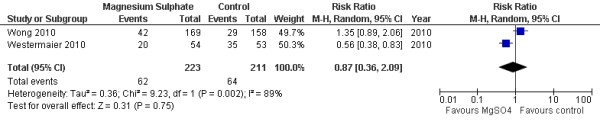
**Random-effects model of risk ratio for delayed cerebral ischemia in aneurysmal subarachnoid hemorrhage patients given magnesium sulphate infusion and a placebo: a comparison **[12,13].

**Figure 2:**
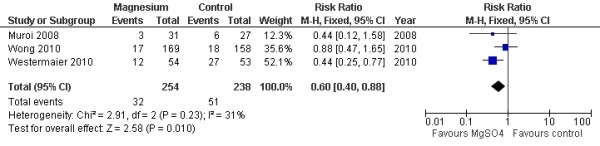
**Random-effects model of risk ratio for delayed cerebral infarction in aneurysmal subarachnoid hemorrhage patients given magnesium sulphate infusion and a placebo: a comparison **[11-13].

Two studies with 434 patients were available for analysis of DCI. The pooled RR for DCI was 0.87 (95% CI = 0.36 to 2.09; *P *= 0.75; Figure 1). Three studies with 492 patients were available for analysis of delayed cerebral infarction. The pooled RR for delayed cerebral infarction was 0.58 (95% CI = 0.35 to 0.97; *P *= 0.04; Figure 2) [15].

**Figure 3:**
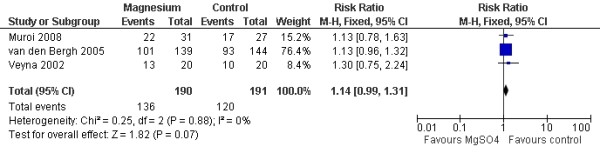
**Fixed-effects model of risk ratio for a favorable outcome at three months: a comparison between magnesium sulphate infusion and a placebo in patients with aneurysmal subarachnoid hemorrhage **[6,11,30].

**Figure 4:**
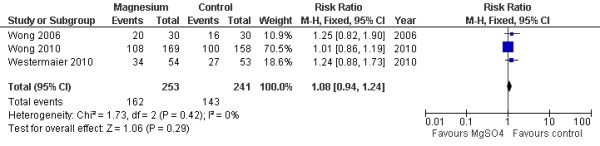
**Fixed-effects model of risk ratio for a favorable outcome at six months: a comparison between magnesium sulphate infusion and a placebo in patients with aneurysmal subarachnoid hemorrhage **[9,12,13].

Three studies with 381 patients and 3 studies with 494 patients were available for analyses of favorable outcome at three months and six months, respectively. The pooled RR for a favorable outcome at three months was 1.14 (95% CI = 0.99 to 1.31; *P *= 0.07; Figure 3), and that for a favorable outcome at six months was 1.08 (95% CI = 0.94 to 1.24; *P *= 0.29; Figure 4).

**Figure 5:**
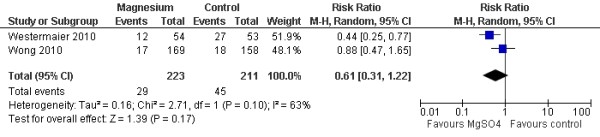
**Random-effects model of risk ratio for delayed cerebral infarction: a comparison between magnesium sulphate infusion and a placebo in patients with aneurysmal subarachnoid hemorrhage (trials with a Jadad score above 3 included) **[12,13].

When only the four high-quality randomized clinical trials (those with a Jadad score above 3) were considered, two studies with 434 patients were available for analysis of DCI. The beneficial effect on delayed cerebral infarction did not persist (RR = 0.61, 95% CI = 0.31 to 1.22; *P *= 0.17; Figure 5), although the other outcomes remained statistically similar.

## Discussion

The results of our up-to-date meta-analysis suggest that the present findings do not lend support to a beneficial effect of magnesium sulphate infusion in reducing DCI and leading to better clinical outcome. Some would argue that these negative findings could be due to a lack of tight neuro-intensive monitoring and treatment, thereby resulting in variation in management outcomes and diluting the neuro-protective effects of magnesium sulphate infusion [24]. However, whether tight parameter control, such as glycemic control, would result in better neurological outcomes remains debatable [24].

The recent meta-analysis by Ma and colleagues suggested that intravenous magnesium therapy reduced the risk of DCI and poor outcome after aneurysmal SAH [18]. However, they have not taken into account the widely varied outcome definitions and time points among studies and simply pooled for assessments. Moreover, two more studies had been published since the meta-analysis by Ma and colleagues. The Asian-Australasian IMASH study was the first international multi-center randomized controlled clinical trial in testing the efficacy of intravenous magnesium sulphate infusion in aneurysmal SAH with negative results in all clinical and surrogate outcomes [12]. Westermaier and colleagues found a significant 29% decrease in delayed cerebral infarction and a nonsignificant 9% decrease in unfavorable outcomes with magnesium sulphate infusion [13]. However, in the landmark British aneurysm nimodipine trial and recently published IMASH report, the differences in the proportions of delayed cerebral infarction paralleled those in the proportions of favorable outcomes [12,25]. Whether these decreases represent a change in the pattern of detected delayed cerebral infarction with the evolution of endovascular treatment and neuro-intensive care remains to be investigated.

There are several limitations to the meta-analysis presented in this paper. The regimens of magnesium sulphate infusion varied across the different studies, and caution should thus be exercised in extrapolating the results. Other problems include the wide variation in the definitions of outcome measures and the time points of assessment, making a smaller number of studies available for meta-analyses of different outcome measures. Furthermore, there may be a publication bias toward the reporting of positive trends and spuriously inflated effects in smaller studies. Statistical heterogeneities were noted in the meta-analyses for DCI and delayed cerebral infarction. The risk of bias was assessed with Jadad criteria [22]; the other option would have been to employ domain-based evaluation [26]. However, any proposed tool would have difficulties to validate, and realistic assessment is eventually open to subjectivity. The recent consensus is that cerebral infarction on plain computed tomography (CT) scan is the most objective measure of DCI, based on the British Aneurysm Nimodipine Trial and other retrospective analyzes [15,5], in which the data were obtained primarily from microsurgically treated intracranial aneurysms. However, identifying delayed cerebral infarction from among all causes of CT hypointensities can be difficult with plain CT alone [27].

Although our *post-hoc *analysis of the IMASH data did not suggest a higher achieved plasma magnesium concentration to be associated with a better clinical outcome [28], the current evidence suggests that the development of a clinical trial targeting reductions in symptomatic vasospasm and cerebral infarction, and an improvement in clinical outcomes, would require a large sample size (as many as 2,445 patients) to demonstrate the efficacy of such treatment in improving neurological outcomes, even with a treatment effect size of 50% [29]. Finally, the relatively small number of patients in the present meta-analysis could mask a possible smaller beneficial effect of magnesium sulphate infusion. Another European multi-center trial (Magnesium in Aneurysmal Subarachnoid Hemorrhage: MASH II) employing a slightly lower dosage regimen is ongoing and should contribute further data for future meta-analysis of the use of magnesium sulphate infusion in patients with aneurysmal SAH [30].

## Conclusions

The reduction in DCI and improvement in clinical outcomes for aneurysmal SAH patients with magnesium sulphate infusion observed in previous pilot studies has not been confirmed although a beneficial effect cannot be ruled out because of sample size limitation.

## Key messages

• Pilot studies have suggested the possible beneficial effects of magnesium sulphate infusion in treating patients with aneurysmal SAH, but previous meta-analyses have become outdated by recently published clinical trials.

• An up-to-date systemic review and meta-analysis showed that magnesium sulphate infusion does not reduce DCI or improve neurological outcomes although a beneficial effect cannot be ruled out because of sample size limitation.

## Abbreviations

CI: confidence interval; CT: computed tomography; DCI: delayed cerebral ischemia; GOS: Glasgow Outcome Scale; IMASH: Intravenous Magnesium Sulphate for Aneurysmal Subarachnoid Hemorrhage trial; MASH: Magnesium and Acetylsalicylic Acid in Subarachnoid Hemorrhage trial; MASH: Magnesium and Acetylsalicylic Acid in Subarachnoid Hemorrhage; mRS: modified Rankin Scale; RR: relative risk; SAH: subarachnoid hemorrhage.

## Competing interests

The authors declare that they have no competing interests.

## Authors' contributions

All of the authors contributed to the design of the study. GKW, SCN, and BZ were responsible for the statistical analysis. GKW drafted the manuscript. All of the authors critically revised the manuscript and agreed on the submitted version.
